# Establishing the relevance of psychological determinants regarding physical activity in people with overweight and obesity

**DOI:** 10.1016/j.ijchp.2021.100250

**Published:** 2021-04-25

**Authors:** Cristina Lugones-Sanchez, Rik Crutzen, Jose I. Recio-Rodriguez, Luis Garcia-Ortiz

**Affiliations:** a Institute of Biomedical Research of Salamanca (IBSAL), Primary Care Research Unit of Salamanca (APISAL), Health Service of Castile and Leon (SACyL), Salamanca, Spain; b Department of Health Promotion, Care and Public Health Research Institute (CAPHRI), Maastricht University, The Netherlands; c Department of Nursing and Physiotherapy, University of Salamanca, Spain; d Department of Biomedical and Diagnostic Sciences, University of Salamanca, Spain

**Keywords:** Physical activity, Accelerometry, Motivation, Obesity, Experiment, Actividad física, Acelerometría, Motivación, Obesidad, Experimento

## Abstract

To identify the most relevant determinants involved in Physical Activity (PA) changes in the EVIDENT 3 study population, measured by the International PA Questionnaire (IPAQ) and the Actigraph GT3X accelerometer.Method: Exploratory study. Data used were collected from EVIDENT 3 study (*N* = 650). Items to measure psychological determinants were chosen from the baseline questionnaires. PA minutes/week were assessed by an accelerometer and IPAQ. The sample was analyzed by the control group (CG), the intervention group (IG) and Body Mass Index, using Confidence Interval-Based Estimation of Relevance (CIBER) analyses. Results: 486 participants, (IG: *n* = 251, CG: *n* = 235) were included. IG shows a positive association between PA assessed by accelerometer and self-efficacy. In IG, the overweight sample shows a positive association between PA assessed by accelerometer and motivation and self-efficacy. PA assessed by accelerometer obtained a higher explained variance (*R^2^*) in IG, both people with overweight (.10 - .55) and obesity (.03 - .19). In CG, IPAQ reached better results in people with overweight (.12 - .49). Conclusions: Motivation and self-efficacy showed as relevant in increasing PA minutes/week, but only in the people with overweight in IG. There might be other factors not analyzed that could improve the low R^2^ obtained.

## Antecedentes/Objetivo

Identificar los determinantes más relevantes en el aumento de la actividad física (AF) de la población incluida en el estudio EVIDENT 3, medido con IPAQ y acelerómetro Actigraph GT3X. Método: Los datos se recogieron del estudio EVIDENT 3 (*N* = 650). Las preguntas para medir los determinantes psicológicos se recogieron de los cuestionarios basales. Los minutos de AF se midieron con el IPAQ y acelerómetro. Se analizó la muestra por grupo de estudio: control (GC) e intervención (GI) y por IMC con el método CIBER (*Confidence Interval-Based Estimation of Relevance*). Resultados: Se incluyeron 486 participantes (GI: *n* = 251, GC: *n* = 235). En GI se mostró asociación positiva entre el acelerómetro y la autoeficacia. Las personas con sobrepeso del GI mostraron asociaciones del acelerómetro con la motivación y la autoeficacia. El acelerómetro obtuvo mayor varianza explicada (*R^2^*) en el GI, tanto en personas con sobrepeso (0,10 – 0,55) como con obesidad (0,03 – 0,19). En el GC el IPAQ obtuvo mejores resultados en personas con sobrepeso (0,12 – 0,49). Conclusión: La motivación y la autoeficacia fueron relevantes para aumentar los minutos de AF, pero solo en personas con sobrepeso del GI. Puede haber factores no incluidos que expliquen el bajo *R^2^* encontrado.

Regular physical activity (PA) is related to a reduction in all-cause mortality and prevention of cardiovascular diseases, type 2 diabetes, hypertension, anxiety and depression (Aggio et al., 2020). However, high levels of inactivity have been reported worldwide over time (Guthold et al., 2018), so promoting regular physical activity remains a public health priority. Engaging in an active lifestyle is a complex behavioral process that is influenced by personal, social and environmental factors (Pan et al., 2009). Levels of physical activity are highest for males, for the young, and for those with higher educational/socioeconomic status (Hallal et al., 2012) except for all types of walking (Pollard & Wagnild, 2017), where women are more willing to do it at any age. This variability between groups shows how various factors are related to PA, so it is crucial to identify which determinants are associated with this behavior. This allows for planning interventions that are capable to target these determinants and, consequently, improve PA level.

There is a great number of determinants that are relevant to behavior, classified as environmental, genetics and psychological variables. This study focuses on psychological variables, as they are most likely to be changeable by an intervention compared with the others, and all environmental and genetic influences on behavior eventually operate through a psychological variable (Crutzen et al., 2017). Previous studies reported there was a positive association between PA on the one hand and enjoyment (Leone & Ward, 2013), expected benefits, intention, perceived health, self-motivation, stage of behavior change, self-schemata for exercise and self-efficacy (Van Dyck et al., 2011) on the other hand. However, research on PA determinants is limited by problems of measurement of activity. Several studies used self-reported questionnaires only and, due to its subjectivity, might give less accurate indications of PA than measurement by accelerometers. Correlations between methods generally were low-to-moderate (Prince et al., 2008), suggesting that non-shared variance among both may lead to differences in PA associations with determinants depending on PA measurement. In line with this, a study (Dishman et al., 1992) concluded that the determinants in adults depend on the type of measurement employed. Therefore, it is warranted to use, more than one type of measurement, because measurement by accelerometers cannot classify domain-specific activity (C. E. Tudor-Locke & Myers, 2001) (e.g., when activity is done for work transport or leisure) while self-reported measurement is likely to include bias (e.g., social desirability and over-reporting) (Sallis & Saelens, 2000). Moreover, determinants’ relevance may vary depending on population. In terms of health promotion, people with chronic diseases are of interest because becoming more physically active could improve their condition notably. Following this line, individuals with obesity are a priority group due to its worldwide prevalence and long-term issues associated with obesity. Despite the lack of strong evidence as to causation (Pazzagli et al., 2019), sedentary behavior itself and a low level of PA are relevant for obesity (Teixeira et al., 2002) and are frequently used as target behaviors in weight loss interventions. Regarding determinants, recovery self-efficacy and social support seem to be associated with PA, but not planning (Parschau et al., 2014). However, comparison between accelerometer and self-reported measurements to set PA determinants relevance in this population have not been explored in-depth.

An appropriate behavior change intervention should include as one of the main goals the modification of determinants related to the behavior of interest. Moreover, selection of determinants by relevance is required because resources to develop the intervention, as well as the time of intervention, participants and staff, is limited. To establish the relevance of determinants two types of analyses need to be combined: assessing the univariate distribution of each determinant and assessing associations to behavior and/or determinants of behavior. This combination is needed because there may be a strong association between a determinant and behavior, but if the distribution is skewed, we would focus only in a subsample, misleading the selection of determinants for the intervention. These analyses are mostly done by computing point estimates (e.g., correlation coefficients or regression coefficients), with some interpretation problems (Crutzen et al., 2017). Thus, it is warranted to base such decisions on confidence intervals (CI) combined with the information about determinant’s distributions and means. Thereby the purpose of the Confidence Interval-Based Estimation of Relevance (CIBER) approach is to combine these metrics (correlation coefficients, means and CI of both) and present them in an understandable way. This visualization facilitates comparison, which is necessary when making selections.

This paper shows the results of an exploratory study using the CIBER approach to identify the most relevant determinants involved in PA short-term (3 months) changes in the EVIDENT 3 study population, measured by the International Physical Activity Questionnaire (IPAQ) and accelerometer. The EVIDENT 3 study data was reused and analyzed by CIBER approach. Due to its exploratory nature, the aim of this study is not to test a specific theory or hypothesis, but identify the most relevant aspects of the members of the target population’s psychology regarding PA behavior.

## Method

### Design

Data used in this exploratory study were collected from the EVIDENT 3 study, where 650 Spanish adults were recruited and included in a randomized, controlled and multicenter trial which involved 5 health care centers from different Spanish regions and it aimed to promote healthy lifestyles to weight loss. At baseline visit, participants in both study arms of EVIDENT 3 study (control and intervention) received 5 minutes of counselling in diet and physical activity prior to randomization. In addition, the intervention group (IG) received a smartphone app and a smartband (Mi Band 2, Xiaomi, China), for 3 months. The EVIDENT application was designed to allow a daily self-reported dietary intake, integrating the data to create specific diet recommendations and weight loss goals. Smartband was used to establish the PA goal of 10,000 steps per day and set sitting time messages. At the 3-month visit, these devices were collected. The investigator who performed the intervention was different from the investigator who conducted the evaluation. The trial was registered at ClinicalTrial.gov with identifier NCT03175614. For this study purpose, data regarding changes in PA from baseline to 3-month visit and determinants at baseline were included in the analyses. Data were collected between June 2017 and November 2019.

The study was approved by the Clinical Research Ethics Committee of Health Area of Salamanca in April 2016. All procedures were performed in accordance with the ethical standards of the institutional research committee and with the 2013 Declaration of Helsinki. All patients signed written informed consent documents prior to participation in the study.

### Participants

**Figure 1:**
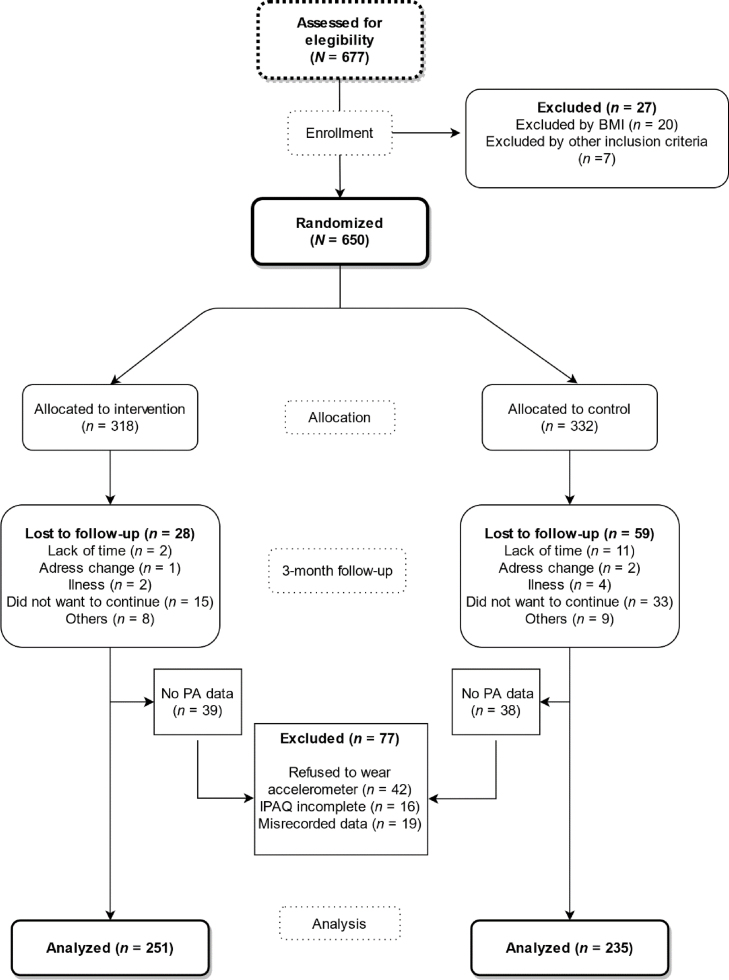
CONSORT flowchart.

All current patients of the 5 health centers were eligible for the EVIDENT 3 study. Each collaborating healthcare professional listed potential participants among the users attending their consultation. Inclusion criteria were sedentary people with BMI 27.5-40 kg/m^2^ between 20-65 years and informed consent signed. Exclusion criteria were type 2 diabetes, neoplasm with active treatment or to be on a diet at baseline visit (Recio-Rodriguez et al., 2018). A researcher of the group identified those who met the criteria described and invite those potential participants by phone and provided the necessary study information. Figure 1.

### Measurements

Sociodemographic data. Trained nurses gathered sociodemographic data at baseline visit, asking age, sex, educational level (illiterate, primary studies, high school, university degree or PhD) and working status (unemployed, student, homemaker, retired or working). In addition, smoking status (non-smoker, former smoker, smoker) and the number of cigarettes were evaluated. Personal history of hypertension, dyslipidemia, and diabetes mellitus was consulted on medical records and verified by the patient at the visit. A detailed information on how the data was collected could be consulted in the study protocol (Recio-Rodriguez et al., 2018).

Anthropometric measures. These values were measured with the subjects barefoot and wearing light clothing. Height was measured twice using a portable system (Seca 222; Medical scale and measurement system, Birmingham, UK). Body weight was measured twice using a calibrated electronic scale (Scale 7830; Soehnle Professional GmbH & Co, Backnang, Germany). Data recorded were the average of the two readings in both cases. Body Mass Index (BMI) was calculated by weight (kg) divided by the height squared (m^2^).

Physical activity Self-report: The short version of the International Physical Activity Questionnaire (IPAQ; Craig et al., 2003) was used to measure PA both at baseline and 3-month visit. The IPAQ is a self-reported questionnaire that provides an estimate of PA time and calculated energy expenditure global and for each activity level: light (walking), moderate and vigorous intensity, showing a reliability of about .65 (*r* = .76; CI 95% = .73 - .77). For each level, participants reported frequencies such as days per week and average duration in minutes over the past week. Accelerometer: The ActiGraph GT3X accelerometer (ActiGraph, Shalimar, FL, USA) was used to measure PA. At the final of both visits (baseline and 3-month follow-up), participants were asked to wear the accelerometer for 7 consecutive days to the right side of the waist throughout the day and to remove it only for water activities (e.g. swimming or bathing). After this period, the device was collected. Data from participants with at least 600 min of wearing time for at least 5 days (including 1 weekend day) were included in the analyses. Non-wearing time was defined as 60 min or more of consecutive zero counts. In both cases, the main outcome of physical exercise was total minutes of PA per week, corresponding to the sum of all PA levels minutes.

Determinants. Items to measure psychological determinants are shown in Table 1, and they were chosen from the baseline questionnaire (Appendix A, Supplementary data): Six self-reported items based on stages of change (Prochaska & Clemente, 1982) evaluated the readiness to change of participants as well as self-efficacy and motivation. All of these items had to be answered by a 5-point Likert scale (α = .67, ω = .69). IWQoL-Lite. The short form of Impact of Weight in Quality of Life is a 31-item, self-report, obesity-specific measure of health-related quality of life (Kolotkin et al., 2001) through assessing five dimensions using a 5-point Likert scale (α = .94, ω = .95). Items of the questionnaires above related to any of the determinants under study were included in the analyses (α = .87, ω = .89).

**Table 1:** Operationalizations and determinants used in the study.

Code	Question	Determinant	Answer categories and coding
M1	How motivated do you feel to lose weight?	Motivation	1. Not motivated, 2.Slightly motivated, 3. Somewhat motivated, 4. Quite motivated, 5. Extremely motivated
M2	Which level of self-confidence do you have to keep going and achieve your goal?	Self-efficacy	1. Not sure, 2. Slightly sure, 3. Somewhat sure, 4, Quite sure, 5. Extremely sure
M3	How likely is it that you can adapt to changes despite them?	Self-efficacy	1. Very unlikely, 2. Somewhat likely, 3. Probable, 4. Quite likely, 5. Extremely likely
A8	I feel short of breath with only mild exertion	Perceiving health values	1. Not true, 2. Rarely true, 3. Sometimes true, 4. Mainly true, 5. Always true
A9	I am troubled by painful or stiff joints	Perceiving health values	1. Not true, 2. Rarely true, 3. Sometimes true, 4. Mainly true, 5. Always true
A11	I am worried about my health	Risk perception	1. Not true, 2. Rarely true, 3. Sometimes true, 4. Mainly true, 5. Always true
B1	Because of my weight I am self-conscious	Self-stem	1. Not true, 2. Rarely true, 3. Sometimes true, 4. Mainly true, 5. Always true
B2	Because of my weight my self-estem is not what it could be	Self-stem	1. Not true, 2. Rarely true, 3. Sometimes true, 4. Mainly true, 5. Always true
B3	Because of my weight I feel unsure of myself	Self-stem	1. Not true, 2. Rarely true, 3. Sometimes true, 4. Mainly true, 5. Always true
B5	Because of my weight I am afraid of being rejected	Social-support	1. Not true, 2. Rarely true, 3. Sometimes true, 4. Mainly true, 5. Always true
B7	Because of my weight I am embarrassed to be seen in public places	Public stress	1. Not true, 2. Rarely true, 3. Sometimes true, 4. Mainly true, 5. Always true
D3	Because of my weight I worry about fitting through aisles or turnstiles	Public stress	1. Not true, 2. Rarely true, 3. Sometimes true, 4. Mainly true, 5. Always true
D5	Because of my weight I experience discrimination by others	Public stress	1. Not true, 2. Rarely true, 3. Sometimes true, 4. Mainly true, 5. Always true
E1	Because of my weight I have trouble getting things accomplished my responsibilities	Work stress	1. Not true, 2. Rarely true, 3. Sometimes true, 4. Mainly true, 5. Always true
E2	Because of my weight I am less productive than I could be	Work stress	1. Not true, 2. Rarely true, 3. Sometimes true, 4. Mainly true, 5. Always true

### Data analysis

Data were input and managed using a REDCap (System Electronic Data Capture; Harris et al., 2019) database. Descriptive analysis was performed by IBM SPSS Statistics v.23 (IBM Corp, Armonk, NY, USA). Measures of central tendency and distribution of study variables were examined at baseline and 3-month visit, as well as tests for normality. The results were expressed as mean and SD for quantitative variables and as frequency distribution for categorical variables. For CIBER analyses, all determinants described above were included. Outcome variables were difference in minutes of PA between baseline and 3-month visit, measured by IPAQ and accelerometer.

The sample was analyzed per study group (CG and IG) to compare the possible differences in relevance of determinants between groups, as IG received an enhancing lifestyles intervention for 3 months while CG was given the common brief advice only. In addition, sub-analyses by BMI, classified as overweight (BMI between 27.5 kg/m^2^ and 30 kg/m^2^) and obesity (BMI ≥ 30 kg/m^2^), were conducted to explore differences, since other studies such as the Diabetes Prevention Program (Delahanty et al., 2002), reported that psychological and behavioral characteristics, including exercise and self-efficacy, were related to baseline BMI.

Reliability analysis of the questionnaires was conducted *by psych package* (Revelle, 2020), CIBER analyses were conducted using the *behavior change package* (Peters, 2021) and R version 3.6.3. In order to foster accurate replication and facilitate future studies, the R script used for the analyses presented in this article are available at Open Science Framework (Lugones-Sanchez & Crutzen, 2021).

## Results

### Sample characteristics

**Figure 2:**
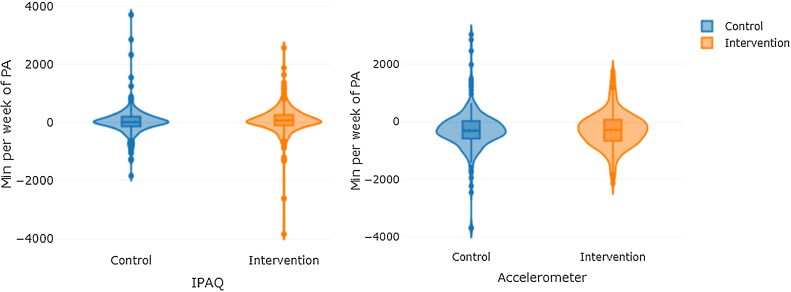
Violin plot of total PA differences distribution by study group measured by IPAQ and accelerometer.

A total of 650 participants were included in the program and randomized to the IG or CG. Loss to follow-up was 13.40% (IG 8.80%, CG 17.70%). Testing at the 3-month visit was completed by 563 (86.60%) participants. Besides the 87 subjects which dropped out during the study, 77 participants were excluded from the analysis because of missing accelerometer data, not reaching a minimum of days registered or because person refused to wear it. Thus, 486 participants, (IG: *n* = 251, CG: *n* = 235) were finally included in the physical activity analyses. The mean age of the entire sample was 47.80 ± 9.71. Both groups had a similar mean age (47.30 ± 9.9 IG and 48.62 ± 9.4 CG) and most participants were women (68.10% and 68.50%, respectively) (Table 2). Mean baseline minutes of physical activity measured by IPAQ was 334.93 ± 336.3 min/week in IG and 328.48 ± 362.3 min/week in CG, while PA measured by accelerometer was 1806.54 ± 626.24 min/week and 1827.50 ± 590.76 min/week respectively in each group (Figure 2). No differences at baseline characteristics were observed between groups.

**Table 2:** Baseline characteristics of the sample.

Baseline characteristics	Intervention group	Control group
	*n* = 251 (51.60%)	*n* = 235 (48.40%)
Age in years, mean (*SD*)	47.30 (9.90)	48.62 (9.40)
Sex (woman), *n* (%)	171 (68.10)	161 (68.50)
Work situation, *n* (%)		
Works outside home	175 (69.70)	177 (75.30)
Homemaker	19 (7.60)	12 (5.10)
Retired	16 (6.40)	8 (3.40)
Student	8 (3.20)	21 (8.90)
Unemployed	33 (13.20)	5 (2.20)
Clinical variables, mean (*SD*)		
Weight (Kg)	91.08 (14.30)	90.94 (14.70)
BMI (Kg/m2)	32.98 (3.30)	32.88 (3.50)
IPAQ, mean (SD)		
PA total min/wk	334.93 (336.30)	328.48 (362.30)
Sedentarism min/wk	2904.86 (1395.90)	2810.10 (1381.20)
Accelerometer, mean (*SD*)		
PA total min/wk	1806.54 (626.24)	1827.50 (590.76)
Sedentarism min/wk	8278.51 (626.63)	8259.71 (585.05)

**Figure 3:**
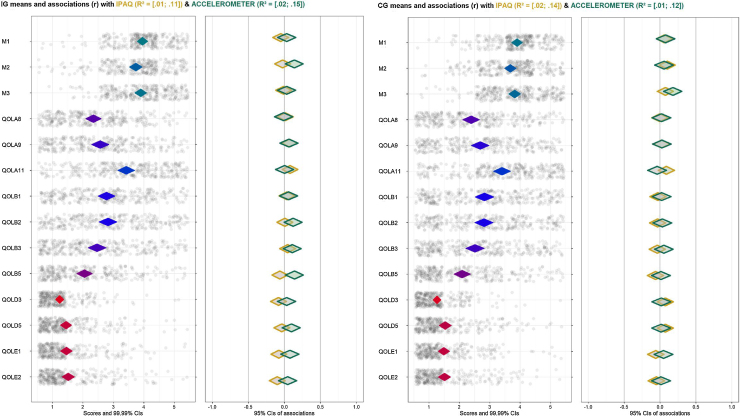
CIBER plot dividing by study group (IG: Intervention group [left], CG: Control group [right]).

Figure 3 shows the output following the proposed analytical approach dividing by study group (CG and IG). The selected items are shown to the left of the left-hand panel. Possible responses to the items, numbered from 1 to 5, are shown below the left-hand panel. The coding of each number can be consulted in Table 1.

**Figure 4:**
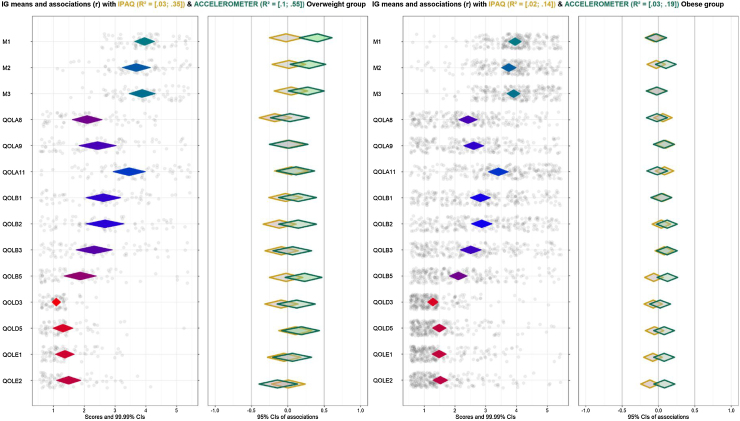
CIBER plots of intervention group (IG) divided by overweight (left) and obese (right).

**Figure 5:**
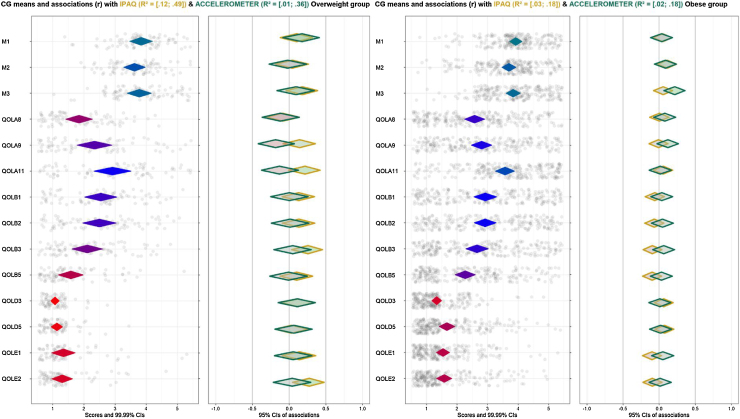
CIBER plots of control group (CG) dividing by overweight (left) and obese (right).

Diamonds for each item show a 99% Cl, and its fill color shows the item means -the redder the diamonds are, the lower the item means (left-skewed); the greener the diamonds are, the higher items means (blue is indicative of means in the middle of the scale). The dots show each participant response to the item with jitter added to prevent overpotting. The diamonds on the right-hand panel show the association strengths (correlation coefficients with 95% CI) between individual items (minutes of PA difference measured by questionnaire and accelerometer in this case) and determinants. The fill color of these diamonds indicates the association strengths and their direction–red diamonds mean strong and negative association, green diamonds strong and positive association and grey means weak association. Diamonds with a yellow stroke show the association with IPAQ and the diamonds with a green stroke show the association with the accelerometer. The CI of the explained variance (*R^2^*) of IPAQ and accelerometer on all determinants are depicted at the top of the figure. Figure 4 depicts the results dividing by BMI –overweight (27.50 kg/m^2^ < BMI < 30 kg/m^2^) and obese (BMI ≥ 30 kg/m^2^)-in the intervention group and Figure 5 in the control group.

### Psychological determinants within groups

The IG shows a positive association between total PA and Self-efficacy (M2) but the scores of this determinant are slightly right-skewed. No association has been found with any other determinant. Otherwise, the CG shows no clear association as expected, with similar distribution in answers. Both groups show an explained variance (*R^2^*) rather low, likely indicating the influence of some characteristics of the population not included in the analyses.

Dividing each group by BMI, the two types of measurements have greater differences in the overweight group than in the obese group. In the intervention group (Figure 4), sample with overweight shows a positive association between PA minutes on one hand and motivation (M1) and self-efficacy (M2) on the other hand with accelerometer only. The distribution of answers is right-skewed but near the middle of the scale, indicating that the majority of people referred to some degree of motivation or self-efficacy to change at baseline. Although distribution is not centered, they might be considered relevant to the target, as influencing positively increased PA after the intervention. However, the obese group shows no remarkable associations nor with IPAQ neither accelerometer, even though the distribution of answers for motivation and self-efficacy items are similar.

While in the control group (Figure 5), strong associations have not been found. However, the results suggest a positive-trend association (but not significative) with self-esteem and work stress in self-reported measure, but not with PA assessed by accelerometers, in the overweight group.

### Determinants with PA measured by accelerometer

The intervention group shows a positive association between total PA and Self-efficacy (M2). Dividing by BMI, motivation (M1) and self-efficacy (M2) show the highest associations with PA in people with overweight, while there are no clear associations with any determinant in the obese group. However, the control group shows no association. There are no strong associations in the overweight group, nevertheless the diamonds trend towards a negative association with health value (A8, A9) and risk perception (A11).

### Determinants with PA measured by self-reports

There are not associations with IPAQ in the IG, trending to the central line in the majority of determinants. Same results can be observed dividing this group by BMI. In the case of the CG, there are also no association with any determinant. Despite the lack of relevant associations, there are positive trends between Self-esteem (B3) and work stress (E2) with PA in the overweight group. Regarding their distribution (trending to left-skewed in both cases) this may suggest that an absence of work stress might be related to a higher level of self-reported PA.

### Explained variance across all behaviors

All figures show a lower *R^2^* than expected, which implies that there are factors involved in the behavior not included in the analyses. Nonetheless, it is worthy to point out the *R^2^* differences between methods. Accelerometer has obtained higher results in the intervention group, both people with overweight [.10, .55] and obesity [.03, .19], while, in the control group, IPAQ reached better results in people with overweight ([.12, .49] and similar to the accelerometer in the obese group [.03, .18]. These discrepancies are likely based on the intervention since IG increased minutes of PA (measured by accelerometer) while CG, knowing the PA recommendations, may over report their PA time at 3-month visit.

## Discussion

The study suggests that the most relevant determinants found in the intervention group to increase PA minutes per week are motivation and self-efficacy, but only in people with overweight. These determinants were relevant to the behavior target only with accelerometer, not finding associations with IPAQ. Our work provides insight in the importance of psychological determinants for increasing PA and differences in relevance between both types of measurements. Despite the trivial effect sizes in terms of correlations, the control group, which received only brief counselling enhancing healthy lifestyles, obtained better-explained variance with IPAQ, revealing the convenience of using both measures in determinants study.

Previous studies have mostly relied on self-report questionnaires to assess the main determinants of the sample. The main problem is the lack of accuracy of this measurement method, due to people overestimate their daily physical activity time, referring to walking time as the least reliably recall (C. E. Tudor-Locke & Myers, 2001). On the other hand, motion sensors have been included in several studies showing a positive association between self-efficacy on weekdays (Maher et al., 2016) and autonomous motivation (Emm-Collison et al., 2020). However, an umbrella review (Cortis et al., 2017) found that psychological determinants have been predominantly analyzed in youth or youth and adults combined and the majority of the studies included assess PA by means of self-reports. These results underline the lack of strong evidence about PA determinants in adults only and the evaluation by accelerometers. Comparison between 2 methods to assess PA was explored in adults before, detecting differences in mean steps per day (C. Tudor-Locke et al., 2002), as well as differences in determinants relevance (Dishman et al., 1992). Moreover, a standardized PA variable that should be used among all the possible (METs, steps/day, PA time) has not been established, hindering comparisons between studies results and drawing definitive conclusions. In our study, the variable used was overall PA time, which appears as the most evaluated (Cortis et al., 2017), finding that mean PA minutes per week differed at baseline between assessments, following the line of previous studies. These differences may be due to people not correctly perceiving their PA or because they may feel observed wearing a motion sensor.

This study was focused on people with overweight and obesity due to its increasing prevalence (Reilly et al., 2018) and the health benefits observed with becoming more active are notorious (Shaw et al., 2006). Exploring PA determinants in this population is needed to lead to lifestyles changes, such as enhancing a higher PA level, instead of focusing on losing weight only. The present study could shed light on this topic, obtaining as relevant determinants motivation and self-efficacy in increasing PA minutes per week, but only in the overweight subsample of the IG. Although replication of these findings in a larger sample is warranted, it could imply that adding a specific goal to increase motivation and self-efficacy in the PA interventions might have a beneficial effect in people with overweight. The results highlight the idea of using various methods to assess PA and analyze associations with determinants, obtaining meaningful information when using them as complementary approaches (Colley et al., 2018, Lipert and Jegier, 2017). Additionally, this paper could improve the knowledge about influences on overall PA changes and enrich future intervention developments.

Although some theories in health psychology could explain the results obtained, the Theory of Planned Behavior (Ajzen, 1991) seems the most appropriate to address the most relevant physical activity determinants found, as has been widely applied to the prediction of health behaviors. In our work, self-efficacy, included as perceived control over PA behavior (Ajzen, 2002), and motivation as indicative of behavioral intention seems to be relevant to explain PA. However, the variance explained is lower than expected, and may be due to other factors not analyzed that may have an influence on the behavior. The extended theory of planned behavior on eating and physical activity (Cheng et al., 2019) includes factors such as weight-related self-stigma (Fung et al., 2020, Pakpour et al., 2019), which may prevent people with obesity from performing PA engagement behaviors as it is highly related to self-efficacy. Future studies will include this factor to evaluate its relevance to address the PA enhancement.

## Limitations

First, data used to this work was obtained from a randomized controlled trial to evaluate an intervention on healthy lifestyles, so questionnaires used to set the possible determinants were not formulated specifically for the study purpose. Despite this fact, the type of the study and the size sample ensure the quality of the results found in this study, as well as the relevance of comparing determinants both types of PA measurements. Based on this paper, further studies could be carried out to address more specifically the main determinants to develop tailored interventions in physical activity to sedentary and people with overweight in the future. Second, the exclusion of people with morbid obesity and those with diabetes, who are prevalent among this population, could limit generalization of the findings. Third, a potential Hawthorne effect (observational bias as a result of being watched) may have also occurred wearing accelerometer. Finally, participants were recommended not to wear accelerometers while swimming or bathing, adding potential measurement bias.

## Conclusions

This exploratory study shows the relevance of the determinants on physical activity among people with overweight and obesity. More specifically, it shows the relevance of motivation and self-efficacy in increasing PA minutes per week, but only in people with overweight in IG. However, there might be other psychologic factors (e.g., attitude, weight-related self-stigma) and barriers not taken into account that could improve the low explained variance with both measures. Some studies referred to socio-environmental determinants (Barnett et al., 2017) as relevant factors in starting and maintaining PA. Further studies will be focused on determining the relevance of factors related to PA in this population with more specific instruments to corroborate the results found as well as exploring the relationship of other PA variables.

This paper also contributes to the existing literature by highlighting the importance of using various methods, types of PA measurements, on the same sample when investigating their relationship with determinants among people with overweight and obesity. The results of the research suggest that future studies should apply both measures of PA when studying psychological variables, as the observed relationships could vary depending on the PA assessment method, not being interchangeable.

## Funding and acknowledgments

The EVIDENT 3 study and the sub-study shown in this paper were funded by the Spanish Ministry of Science and Innovation, Instituto de Salud Carlos III and co-funded by European Union (ERDF/ESF, “Investing in your future”) (PI16/00101, PI16/00952, PI16/00765, PI16/00659, PI16/00421, PI16/00170, FI17/00040, MV19/000001). Gerencia Regional de Salud de Castilla y León (GRS 1277/B/16) also collaborated in the founding of the study. They played no role in the study design, data analysis, reporting results or the decision to submit the manuscript for publication.

We are very grateful to all participants who took part in the EVIDENT 3 study, as well as to the EVIDENT 3 research group for facilitating the data of the study to this purpose. The EVIDENT 3 researchers list could be consulted on apisal.es/investigadores.
