# Supplementary material for: Establishing the relevance of psychological determinants regarding physical activity in people with overweight and obesity
Source: Int J Clin Health Psychol. 2021 Apr 25;21(3):100250. doi: 10.1016/j.ijchp.2021.100250 (PMC8093885; doi:10.1016/j.ijchp.2021.100250)
Supplement: Supplementary file 1 [file mmc1.docx]

**READINESS TO CHANGE**

A.1 How motivated do you feel to lose weight?

- Not motivated
- Slightly motivated
- Somewhat motivated
- Quite motivated
- Extremely motivated

A.2. Which level of self-confidence do you have to keep going and achieve your goal?

- Not sure
- Slightly sure
- Somewhat sure
- Quite sure
- Extremely sure

A.3. Think of all things that are in your life currently (work stress, family duties, etc) How likely is it that you can adapt to your diet despite them?

- Very improbable
- Somewhat likely
- Probable
- Quite likely
- Extremely likely

A.4. Think honestly about how much weight you expect to lose and how quickly you expect to lose it. The mean loss weight is around 0.5-1 kg per week. Would you be satisfied losing 0.5-1 kg per week?

- Barely satisfied
- Poorly satisfied
- Satisfied
- Quite satisfied
- Very satisfied

A.5. When you are on a diet, do you fantasize about eating your favourite dishes?

- Always
- Frequently
- Occasionally
- Rarely
- Never

A.6. When you are on a diet, do you feel deprived of something, angry and/or upset?

- Always
- Frequently
- Occasionally
- Rarely
- Never

**IMPACT OF WEIGHT ON QUALITY OF LIFE (IQWOL-Lite)**

*Physical funtion*

1. Because of my weight i have trouble picking up objects
2. Because of my weight I have trouble tying my shoes
3. Because of my weight I have difficulty getting up from chairs
4. Because of my weight I have trouble using stairs
5. Because of my weight I have difficulty putting on or taking off my clothing
6. Because of my weight I have trouble with mobility
7. Because of my weight I have trouble crossing my legs
8. I feel short of breath with only mild exertion
9. I am troubled by painful or stiff joints
10. My ankles and lower legs are swollen at the end of the day
11. I am worried about my health

*Self-esteem*

1. Because of my weight I am self-conscious
2. Because of my weight my self esteem is not what it could be
3. Because of my weight I feel unsure of myself
4. Because of my weight I don’t like myself
5. Because of my weight I am afraid of being rejected
6. Because of my weight I avoid looking in mirrors of seeing myself in photographs
7. Because of my weight I am embarrassed to be seen in public places

*Sexual activity*

1. Because of my weight I do not enjoy sexual activity
2. Because of my weight I have little or no sexual desire
3. Because of my weight I have difficulty with sexual performance
4. Because of my weight I avoid sexual encounters whenever possible

*Public distress*

1. Because of my weight I experience ridicule, teasing or unwanted attention
2. Because of my weight I worry about fitting into seats in public places (e.g. theaters, restaurants, cars or airplanes)
3. Because of my weight I worry about fitting through aisles or turnstiles
4. Because of my weight I worry about finding chairs that are strong, enough to hold my weight
5. Because of my weight I experience discrimination by others

*Work*

1. Because of my weight I have trouble getting things accomplished or meeting my responsabilities
2. Because of my weight I am less productive than I could be
3. Because of my weight I don’t receive appropiate raises, promotion or recognition at work.
4. Because of my weight I am afraid to go on job interviews

| Always true | Mainly true | Sometimes true | Rarely true | Not true |
| --- | --- | --- | --- | --- |
| 5 | 4 | 3 | 2 | 1 |
